# Native Pig Neutrophil Products: Insights into Their Antimicrobial Activity

**DOI:** 10.3390/microorganisms11082119

**Published:** 2023-08-20

**Authors:** Eric Fernández-De La Cruz, Joanna Wessely-Szponder, Miguel Viñas, Teresa Vinuesa, Alexandra Merlos, Marta Jorba, Paula Espinal, Ester Fusté

**Affiliations:** 1 Laboratory of Molecular Microbiology & Antimicrobials, Department of Pathology & Experimental Therapeutics, Faculty of Medicine & Health Sciences, IDIBELL-University of Barcelona, Campus Bellvitge, 08907 L’Hospitalet de Llobregat, Spain; eric.fernandez@ub.edu (E.F.-D.L.C.); mvinyas@ub.edu (M.V.); tvinuesa@ub.edu (T.V.); amerlig_@hotmail.com (A.M.); m.jorba.pedrosa@gmail.com (M.J.); 2 Sub-Department of Pathophysiology, Department of Preclinical Veterinary Sciences, Faculty of Veterinary Medicine, University of Life Sciences, 20-950 Lublin, Poland; joanna.wessely@up.lublin.pl; 3 Department of Public Health, Mental Health and Maternal and Child Nursing, University of Barcelona, Campus Bellvitge, 08907 L’Hospitalet de Llobregat, Spain

**Keywords:** antimicrobial peptides, cathelicidins, efflux pumps, black lipid bilayer, membrane disruption, atomic force microscopy, transmission electron microscopy

## Abstract

Cationic antimicrobial peptides are molecules with potential applications for treating infections due to their antimicrobial and immunomodulatory properties. The aim of this work was to explore the antimicrobial activity and mechanisms of action of a porcine neutrophil cathelicidin mixture (MPPN). Gram-positive and Gram-negative bacteria were used to determine the minimum inhibitory concentration (MIC) and experiments of both time–kill kinetics and effects on growth curves were performed. Planar black lipid bilayer conductance was measured to analyze the interaction of MPPN with lipid bilayers. Visualization of bacterial surfaces and membrane alterations was achieved using atomic force microscopy and transmission electron microscopy. The effects on the activity of efflux pumps (EPs) were studied with an intracellular accumulation of acridine orange (AO) assay. In *E. coli*, MPPN behaves as a bactericide at high concentrations and as a bacteriostatic at lower concentrations. The bacteriostatic effect was also observed for slightly shorter periods in *S. enterica*. The mixture was not active on *S. aureus.* The increase in AO accumulation in the presence of MPPN indicates that, at least in *E. coli*, the mixture causes inhibition of the EP function. Observed and detected variable conductance events demonstrate a strong MPPN effect on lipid bilayers. Damage to the structure of treated *E. coli* indicates that MPPN induces alterations in the bacterial surface. The use of AMPs capable of inhibiting EP can be seen as a good tool to combat antimicrobial resistance since they could be used alone or in combination with other conventional antibiotics to which bacteria have become resistant.

## 1. Introduction

The increase in bacterial resistance to antimicrobials is a relevant threat to public health, and the treatment of infections caused by multidrug-resistant bacteria (MDR) is a pressing need. Among the essential objectives of research in this field is the search for new antimicrobial agents. Antimicrobial peptides (AMPs) (natural and synthetic) have emerged as potential candidates and constitute an inexhaustible source of new antimicrobial molecules [1,2]. Some of these molecules, present in most organisms as an important part of the immune innate response [3], play key roles in cellular differentiation processes, being the first defense line against a large number of pathogens. Natural peptides are also involved in physiological processes such as angiogenesis, cellular signaling and inflammatory responses [4,5]. In terms of their antimicrobial capabilities, many AMPs exhibit broad-spectrum antimicrobial activity against bacteria, fungi and viruses [6,7]. Bo et al. [8] described broad-spectrum antimicrobial activity against relevant aquatic pathogens of naturally extracted peptides that are part of the innate immune system of the rockfish. Dong et al. [9] produced protegrin-1-based AMPs and found that they were active against Gram-negative bacteria such as *Escherichia coli* and *Salmonella typhimurium* and against Gram-positive bacteria such as *Staphylococcus aureus* and *Enterococcus faecalis*.

The most common mechanisms of action of AMPs comprise the disruption of the membrane permeability barrier and entrance into the cytoplasm, reaching targets and inhibiting several intracellular functions [6].

Cathelicidins are small, cationic host-defense peptides displaying antimicrobial and immunomodulatory functions. Some cathelicidins may act by increasing or decreasing the immune response, including activity on neutrophils, where they may modify neutrophil degranulation or regulate the inflammatory response [10]. Cathelicidins consist of a short signal peptide, plus a highly conserved cathelin-like domain, and an active peptide, usually consisting of fewer than 40 amino acids, with a total of four exons involved [11,12].

Cathelicidins are part of the innate immune system of several mammals, including pigs. They are regarded as very useful animals in human medicine and are used not only in xenotransplantation but also as sources of insulin; ACTH from the porcine pituitary gland, a hormone used in human medicine for the treatment of arthritis and inflammatory diseases; porcine thyroids; heparin; human hemoglobin produced by transgenic pigs; and many others [13]. In addition to the known myeloid antimicrobial peptides PMAP-23, PMAP-36 and PMAP-3, there are also porcine cathelicidins extracted from neutrophils, such as proline-phenylalanine-rich prophenin-1 (PF-1) and prophenin-2 (PF-2), proline-arginine-rich 39-amino acid peptide (PR-39) and cysteine-rich protegrin-1-3 (PG-1-3) [14]. Prophenins 1 and 2 from porcine neutrophils are composed of approximately 79–80 amino acid residues with a high content of prolines (53.2%) and a considerable amount of phenylalanine (19%) and arginine (7.6%) [15]. Cathelicidins exhibit killing activity against both Gram-positive and Gram-negative bacteria. Nevertheless, in the case of *E. coli*, the extent of membrane permeabilization does not align with the observed bactericidal capability. This may be observed in living bacteria and in model membranes and has been attributed to membrane depolarization causing disruption of lipid chain packing, which plays a crucial role in the bactericidal activity of the peptides. Many of these peptides are known to kill microorganisms by translocating across membranes and interacting with their nucleic acids. These two categories of membrane-based and intracellular-target-based attack seem to be general in all natural antimicrobial peptides. Prophenin exhibits antimicrobial activity [14], acting as an immunomodulator, where it was observed in rabbit neutrophil degranulation, survival, and apoptosis [16]. PR-39 is a linear cathelicidin made up of 39 amino acid residues, also with a high content of proline (49%) and arginine (26%) [17]. PR-39 is mainly active on Gram-negative bacteria and is bactericidal due to its ability to translocate across the membrane and inhibit the protein and DNA synthesis [17,18,19]. Finally, protegrins 1–3 are cathelicidins that contain 16–18 amino acid residues. They possess four conserved cysteines that form two intramolecular disulfide bonds (between C1–C2 and C3–C4), which confer stability to the antiparallel β-sheet structure [15]. Among these, protegrin 1, isolated from porcine leukocytes, has been shown to have broad antimicrobial activity and potential therapeutic use [20].

Naturally occurring cathelicidins from neutrophil extracts can be used for analysis and have possible therapeutic potential [21,22]. Stimulation of innate immunity without induction or suppression of the harmful pro-inflammatory response appears to be an alternative, as immunomodulators can act on host cells rather than directly attacking bacteria, thereby avoiding the emergence of bacterial resistance and providing an alternative for the control of infections caused by multidrug-resistant bacteria [23,24].

Herein, we explore the antimicrobial activity and the mechanisms of action of a mixture of natural peptides belonging to the cathelicidin family, directly obtained from porcine neutrophils (MPPN), as previously described by Wessely et al. [14].

MPPN displayed significant antimicrobial activity on *E. coli* ATCC 25922 and *S. enterica* ATCC 13076, but not on *S. aureus* ATCC 29313. MPPN induced morphology alterations on the surface of *E. coli*, caused inhibition of the efflux pumps’ function, and altered the conductance and stability of artificial lipid membranes even through formation of pores, suggesting that the mechanisms of action of MPPN involve membranes and inhibition of the efflux pumps.

## 2. Materials and Methods

### 2.1. Strains and Peptides

*Escherichia coli* ATCC 25922, *Staphylococcus aureus* ATCC 29213, *Pseudomonas aeruginosa* ATCC 27583, *Enterococcus faecalis* ATCC 29212, *Acinetobacter baumannii* ATCC 17978, *Klebsiella pneumoniae* ATCC 28,331 and *Salmonella enterica* ATCC 13,076 were used for evaluation of the antibacterial activity of MPPN. All reference strains were provided by the American Type Culture Collection (https://www.atcc.org/ (accessed on 12 June 2023)). Strains were subcultured and aliquots stored at −80 °C in TSB + 10% glycerol.

A mixture of natural peptides from a crude extract of porcine neutrophils was obtained according to the method described by Wessely-Szponder et al. [13] and then lyophilized and stored at −80 °C. Peptides present in the mixture were identified using MALDI-TOF MS analysis (RTOF MS—built in the Institute of Physics, Division of Molecular Physics, UMCS Lublin, Poland, with an ion source MALDI). Parameters were previously described by Głuch et al., 2001 and Gruszecka et al., 2008 [25,26]. Table 1 details the structure, sequences and size of the peptides [15].

### 2.2. Minimal Inhibitory Concentration (MIC) and Minimal Bactericidal Concentration (MBC)

The antimicrobial activity of MPPN was initially evaluated by determining the MIC using the reference broth microdilution method in 96-well microtiter plates as recommended by CLSI [27]. In addition, the MBC of MPPN was determined by plating 100 μL from each well where no visible growth on tryptone soy agar (TSA) (Condalab, Madrid, Spain) was observed. Plates were incubated at 37 °C for 24 h. All experiments were performed in triplicate.

### 2.3. Growth Curves

A starting inoculum of 1 × 10^6^ CFU/mL was used to determine the effect of MPPN on the growth. Briefly, MPPN at MIC and 1/2 MIC was added to cultures in cation-adjusted Mueller–Hinton (CAMHB) and incubated in real-time reverse spin bioreactors RTS-1 (Biosan SIA, Riga, Latvia) at 37 °C for 24 h. Growth was measured non-invasively at an optical density of 850 nm every 15 min for 24 h. All measurements were performed in triplicate.

### 2.4. Time–Kill Curves

MPPN at concentrations identical to the determined MIC value and 1/2 MIC was added to the bacterial cultures (1 × 10^6^ CFU/mL) and incubated for 24 h at 37 °C, with shaking at 200 rpm. Samples were aseptically obtained at 0, 1, 2, 4, 6 and 8 h, serially diluted in Ringer 1/4 and plated on TSA for colony counting. Plates were incubated for 24 h at 37 °C. The response of the strains to MPPN was determined based on a logarithmic decrease in viable bacteria. Time–kill assays were performed in triplicate.

### 2.5. Acridine Orange Intracellular Accumulation

To investigate the inhibition of the efflux machinery in the presence of different concentrations of MPPN, the accumulation of acridine orange (AO) in bacteria was calculated as previously described by Armengol et al. [28]. Briefly, assays were performed in 96-well flat-bottomed microtiter plates. Overnight cultures were diluted in Ringer 1/4 solution to an OD_520nm_ of 1.5 and 100 µL was added to the wells previously filled with 100 µL of Ringer’s solution. AO at 0.25 μg/mL was added and then the inoculated plates were incubated for 1 h with shaking, after which the fluorescence was measured in a FLUOstar OPTIMA fluorescence microplate reader (BMG Labtech, Ortenberg, Germany). The AO uptake was determined in the presence of 20 μg/mL of the efflux pump inhibitor PaβN, in the presence of sub-inhibitory concentrations of MPPN (1/2 MIC, 1/4 MIC, 1/8 MIC), and untreated bacteria was used as the control. The results were expressed as the percentage increase in fluorescence with respect to the control. All experiments were performed in triplicate.

### 2.6. Planar Lipid Bilayer Assays

To analyze a possible interaction of the peptides with lipid bilayers, artificial membranes were reconstituted from 1% (*w*/*v*) diphytanoylphosphatidylcholine (DiPhPC), a zwitterionic lipid, in n-decane. The bilayers were painted over a 0.8 mm diameter hole in a Teflon partition separating two compartments containing 5 mL each of 1 M KCl. Voltages were applied across this membrane via Ag/AgCl electrodes connected by a salt bridge, and the resulting current was amplified 10^9^-fold by a current amplifier and recorded on a Rikadenki R-01 strip chart recorder [29,30].

### 2.7. Atomic Force Microscopy (AFM)

AFM was used to visualize the bacterial surfaces of untreated and treated strains after exposure to MPPN at the MIC and 1/2 MIC. Bacteria were collected by centrifugation at 2000× *g* for 3 min and the pellet was resuspended in sterile water. A volume of 10 μL was applied to the MICA surface, dried at room temperature and then imaged in air by using an atomic force microscope XE-70 (Park Systems, Korea) [31]. All images were collected in non-contact mode using pyramidal-shaped silicon cantilevers with a spring constant of ±40 N/m and a resonance frequency of ±300 kHz, with the upper side coated with aluminum to increase the reflectivity of the laser beam [32]. Data collected during the surface scanning were converted into topography and amplitude images and analyzed using XEP and XEI software version 1.8.0 (Park Systems, Korea) to analyze the shape, structure and surface of the bacteria. AFM images were acquired with a scan size of 25 μm^2^ at a scan rate of 0.3–0.6 Hz [33].

### 2.8. Transmission Electron Microscopy (TEM)

TEM was used to analyze the phenotypic alterations of the treated and untreated *E. coli* with MIC and 1/2 MIC of MPPN. First, *E. coli* in the exponential phase was incubated with the different concentrations of MPPN for 4 h at 37 °C. The cultures were then centrifuged at 2000× *g* for 5 min (HERAEUS BioFuge fresco, Kendro Laboratory Products, Germany) and the pellet was fixed with 2.5% glutaraldehyde and 2% paraformaldehyde in 0.1 M phosphate buffer for 30 min at room temperature. The fixed samples were centrifuged at 2000× *g* for 5 min and the pellet was resuspended in the fixing solution and stored at 4 °C. Bacteria were postfixed with osmium tetroxide (Sigma-Aldrich, Schnelldorf, Germany), dehydrated with acetone (Panreac, Barcelona, Spain), embedded in resin and sectioned with an ultramicrotome (Ultramicrotome UC7, Leica, Viena, Austria). Ultrathin sections (50–70 nm) were stained with 2% uranyl acetate (Thermo Fisher Scientific, Waltham, MA, USA) for 10 min, a lead staining solution for 5 min and finally analyzed on a JEOL 1010 transmission electron microscope with a CCD Orius digital camera (Gatan) (400 kV of maximum TEM operating voltage; CCD active area 15 × 15 mm; readout speed 30/5 MHz; frame rate of ≤30 frames per second and exposure setting of 0.001–100 s).

## 3. Results

### 3.1. Antimicrobial Activity (MIC and MBC)

MPPN had an MIC of 68.7 μg/mL in *E. coli* and *S. enterica,* while higher MICs (>275 μg/mL) were found in *P. aeruginosa*, *A. baumannii*, *K. pneumoniae*, *E. faecalis* and *S. aureus.* The MBC was >275 μg/mL for all strains, except for *E. coli* (137 μg/mL). Results are shown in Table 2.

### 3.2. Effect of MPPN on the Microbial Growth

**Figure 1:**
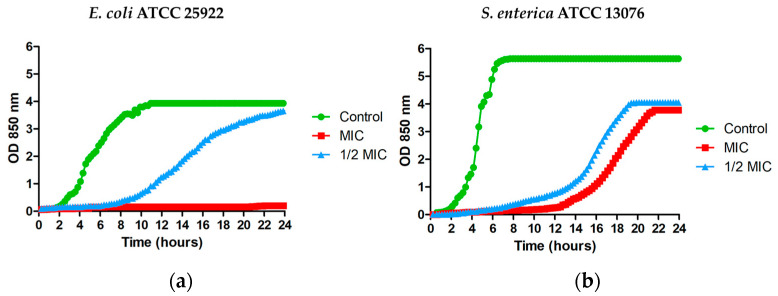
Growth curves of (**a**) *E. coli* ATCC 25922 and (**b**) *S. enterica* ATCC 13076 treated with 68.7 μg/mL (MIC) (red line), 34.4 μg/mL (1/2 MIC) (blue line). Control without MPPN (green line).

As can be seen in Figure 1a, at 68.7 μg/mL (the MIC value), MPPN completely prevented the growth of *E. coli* for 24 h. At 34.4 μg/mL (1/2 MIC), detectable bacterial growth was fully inhibited for six hours. In *S. enterica*, MPPN inhibited growth for twelve hours at 68.7 μg/mL (MIC), and for six hours at 34.4 μg/mL (1/2 MIC) (Figure 1b).

### 3.3. Time–Kill Curves

**Figure 2:**
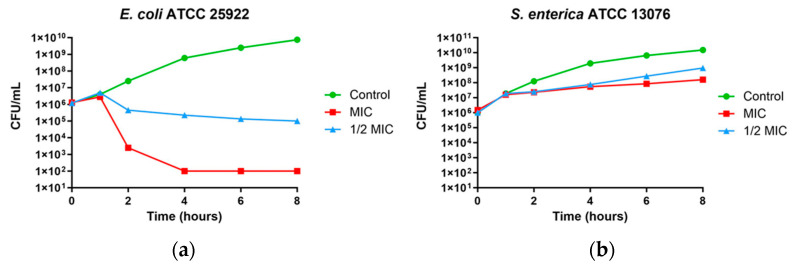
Time–kill curves. (**a**) *E. coli* ATCC 25922 and (**b**) *S. enterica* ATCC 13076 treated with 68.7 μg/mL (MIC) (red line) or 34.4 μg/mL (1/2 MIC) (blue line). Control without MPPN (green line).

In *E. coli* at the MIC, a reduction of 4 logs was observed and was maintained for up to eight hours. At 1/2 MIC, a reduction of approximately one log was observed after two hours and was maintained up to eight hours (Figure 2a). The effect on *S. enterica* at both MIC and 1/2 MIC is shown in Figure 2b.

### 3.4. Acridine Orange Intracellular Accumulation

**Figure 3:**
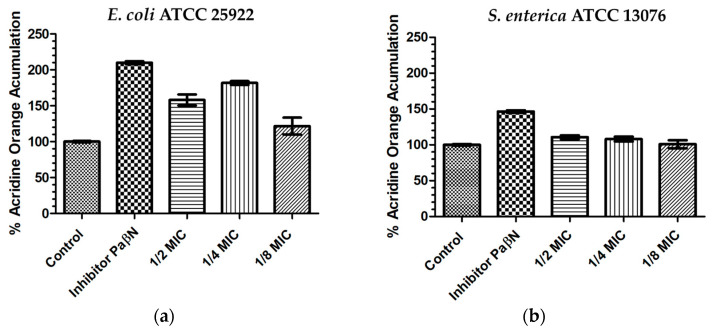
Percentage of acridine orange (AO) accumulation in the presence of the efflux inhibitor PaβN and sub-inhibitory concentrations of MPPN in *E. coli* (**a**) and in *S. enterica* (**b**). Control: bacteria plus AO.

As AO is a good substrate for efflux pumps, and is a well-known resistance mechanism; its intracellular accumulation is enhanced by the inhibition of efflux pumps. Changes in AO accumulation in the presence of MPPN by both *E. coli* and *S. enterica* are shown in Figure 3. In this experiment, the control (untreated bacteria) was considered as 100% AO accumulation. In the experiments performed in *E. coli*, the presence of the efflux pump inhibitor PaβN caused an increase in AO accumulation by nearly 200%; MPPN at sub-inhibitory concentrations led to increases in AO accumulation by nearly 160% at 1/2 MIC, 180% at 1/4 MIC and 120% at 1/8 MIC (Figure 3a). Statistical significance (*p* < 0.05) between the inhibitor PaβN and 1/2 MIC, 1/4 MIC, and 1/8 MIC; and between the control and 1/2 MIC, 1/4 MIC, and 1/8 MIC was found.

In *S. enterica*, the presence of PaβN increased AO accumulation by nearly 150%, whereas MPPN (1/2 MIC and 1/4 MIC) slightly increased AO accumulation (110%). At 1/8 MIC, AO accumulation was equal to the control amount (Figure 3b). Data from the AO accumulation experiments are shown as Supplementary Material.

### 3.5. Planar Lipid Bilayer Assays

**Figure 4:**
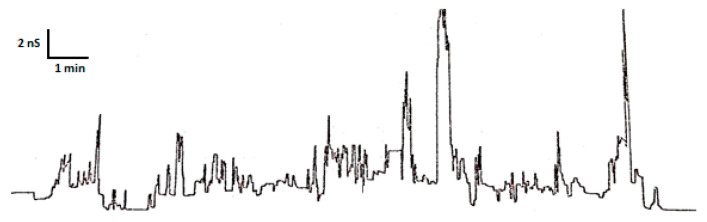
Records of the membrane conductance events that occurred upon addition of MPPN to the solution (1 M KCl) bathing a black planar lipid bilayer at a voltage of −50 mV. Concentration of 41.13 ng/mL. The scale to interpret the registers is represented as time (minutes) on the *x*-axis and conductance (nS) on the *y*-axis.

MPPNs were investigated for their ability to cause conductance events in an artificial planar lipid bilayer. The initial purpose was to explore if they were able to form transmembrane channels. As expected, and shown in Figure 4, some membrane conductance events were observed upon addition of MPPN at −50 mV. Furthermore, many of the events observed resulted in rapid conductance changes of variable magnitude and duration, often with short lifetimes followed by a return to baseline. The observed changes in membrane conductance suggest that MPPNs are membrane-active antimicrobial peptides but that they produce small transient transmembrane channels whose life is short. Moreover, longer lifetimes occurred only occasionally. In contrast, no electrophysiological events were observed at +50 mV. This result is consistent with the positive charge of the peptides. In fact, all transmembrane conductance events were often completely lost when the voltage was switched to positive.

### 3.6. Atomic Force Microscopy (AFM)

**Figure 5:**
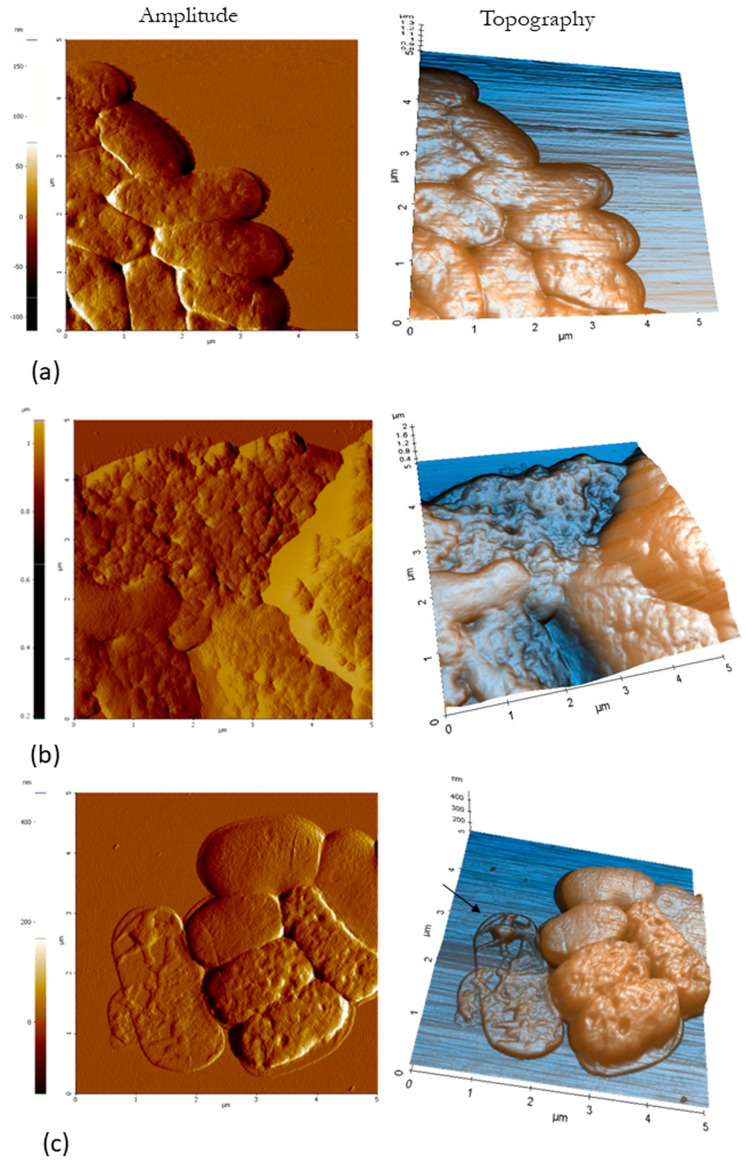
Amplitude and topography AFM images of *E. coli* obtained at a 25 μm^2^ scan size: (**a**) untreated *E. coli*, (**b**) *E. coli* treated at MIC (68.7 μg/mL) of MPPN, and (**c**) *E. coli* treated with 1/2 MIC (34.4 μg/mL) of MPPN. Black arrow indicates a ghost bacterium.

Amplitude and topography AFM images obtained after treatment of *E. coli* with MPPN at MIC, 1/2 MIC and untreated bacteria are shown in Figure 5. Untreated bacteria appeared as typically rod-shaped (Figure 5a), whereas MPPN-treated bacteria showed clearly visible morphological changes. Damage to the overall structure of most MPPN- treated *E. coli* with leakage of cell contents was observed after exposure (Figure 5b,c).

### 3.7. Transmission Electron Microscopy (TEM)

**Figure 6:**
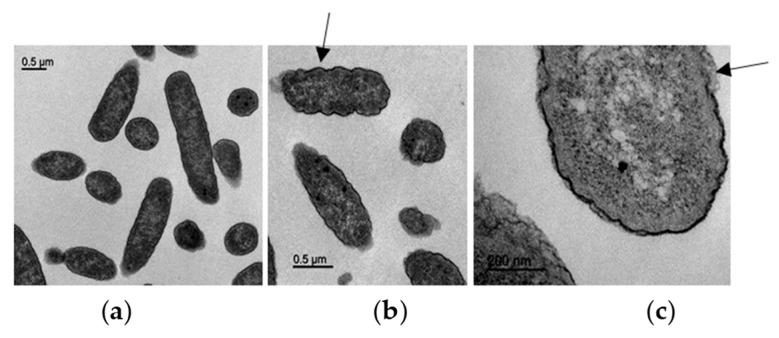
TEM images of the *E. coli* ATCC 25922 (**a**) untreated and (**b**,**c**) treated at the MIC of MPPN. In (**b**), the arrow indicates a waved outer membrane and, in (**c**), the arrow indicates cell envelope disruptions.

TEM observations of ultrathin segments of *E. coli* are shown in Figure 6. Untreated *E. coli* showed the typical bacterial shape without structural damage (Figure 6a). A waved outer membrane and some cell envelope disruptions were detected in bacterial cells after treatment of *E. coli* at the MIC (Figure 6b,c).

## 4. Discussion

Traditional antibiotics are becoming less effective against many bacterial infections due to the growing emergence of multidrug-resistant bacteria, leading to increased morbidity, mortality, and healthcare costs [34]. In this context, the discovery of new therapeutic agents is a worldwide necessity.

Several studies have confirmed that cationic antimicrobial peptides, such as cathelicidins, kill or inhibit microorganisms [35]. Among them, porcine cathelicidins from neutrophils have mainly been studied individually against Gram-positive and Gram-negative bacteria [9,14,36]. In addition, Scapinello et al. [37] found antimicrobial activity of unfractioned porcine neutrophil secretions against relevant swine pathogens (*Actinobacillus suis*, *Streptococcus suis* and *Pasteurella multocida*). Thus, the aim of this work was to explore the antimicrobial activity and mechanisms of action of a mixture of cathelicidins extracted from porcine neutrophils using some human pathogens of clinical relevance. The antimicrobial activity of the mixture of cathelicidins (MPPN) was better, albeit low if one considers MIC values, against the Gram-negative bacteria *E. coli* and *S. enterica* than against the Gram-positive bacteria *E. faecalis* and *S. aureus*. The MIC determination showed values that should be considered elevated (>64 µg/mL). However, MPPN showed good results when considering other ways of evaluating antimicrobial activity, particularly in *E. coli*. MPPN behaved as a bactericide at the MIC and as a bacteriostatic at lower concentrations. The bacteriostatic effect was observed for slightly shorter periods in *S. enterica*. The ability of the peptide mixture to inhibit growth and kill bacteria for limited periods of time at these concentrations may still be relevant, as it could prevent bacterial proliferation for periods of time long enough to confer advantages to the immune system to clear the infection [38]. The results of the time–kill curves confirmed the initial interpretations, since in *E. coli* at the MIC, the product behaves as an effective bactericide, whereas at lower concentrations (1/2 MIC) or in *S. enterica*, it behaves as a bacteriostatic. Previous studies have reported that individual cathelicidins such as prophenin 1 and 2 [39,40]; the antibacterial protein PR-39 [41]; and protegrin 1, 2 and 3 [42,43,44] are effective as microbicides, mainly against *E. coli.* Despite the lack of experimental evidence, a plausible reason may reside in the fact that Gram-negative bacteria are protected from their environment by an outer membrane that is primarily composed of lipopolysaccharides (LPSs). Under stress, pathogenic serotypes of *Salmonella enterica* remodel their LPSs through the PhoPQ two-component regulatory system, which has been shown to determine resistance to both conventional antibiotics and antimicrobial peptides (AMPs), including colistin [45].

The increase in AO accumulation in the presence of MPPN strongly indicates that, at least in *E. coli*, the mixture causes an inhibition of the efflux pumps’ function. This is not the first report of this, since it has been demonstrated that other cationic peptides may exert a similar effect [28]. Drug efflux is a common mechanism of antibiotic resistance, particularly in Gram-negative bacteria, which allows bacteria to extrude harmful substances, such as antimicrobial agents, to keep bacterial concentrations below toxic levels [46,47]. A paradoxical result was obtained as MPPN at 1/4 MIC was more active than at twice the concentration. Despite this, we lack experimental data; this may be due to the fraction of killed bacteria at 1/2 MIC that is not caused by lower concentrations. However, some other reason may not be ruled out.

Despite this, efflux pumps have been shown to be active in extruding antimicrobial peptides, although they do not have any critical role in the tested pathogens as a strategy to increase virulence by circumventing the antimicrobial action of innate defense. Our results demonstrate that (at least in *E. coli*) the peptide mixture may effectively inhibit the efflux machinery. Efflux pump inhibitors increase the cellular concentration of antibiotics to potentiate their function and should be regarded as a very interesting source of novel antibiotic adjuvant scaffolds, which may revert the effects of multidrug resistance.

Another approach to better understand the mechanism of action of novel peptides is to examine membrane damage by electrophysiological experiments. The variable conductance increments, both in magnitude and lifetime, observed in our study demonstrated the ability of MPPN to act on lipid membranes, including the induction of unstable and variable pore formation. Cationic peptides interact with membranes; this is simply derived from their cationic nature. Some of them have been studied using similar or identical methods, such as colistin [48], gramicidin S and bactenecin [30]. In all of these cases, AMPs interact with membranes, causing permeability alterations and sometimes membrane disruption. Despite this, black lipid bilayer conductance measurements are probably not the best manner to study this. The variability of conductance events observed in the present work demonstrates a strong effect of the peptide mixture with the bilayers, although we cannot rule out that some pore-forming molecule may be present in the MPPN, or that some aggregates may have such an effect [49]. This observation also suggests that different cationic peptide molecules could intercalate into the membrane, as has been previously described for peptide NK-lysin [50], CAP18-derived peptides [51] and eumenitin [52].

Since some peptides induce changes in the bacterial surface, AFM studies allowed the visualization of the changes induced by MPPN-treated bacteria. Damage to the overall structure, leakage of cell contents, increased roughness and presence of empty ghost cells were observed in treated *E. coli*. These results indicate that MPPN induces bacterial surface alterations. Although it is not identical, this phenomenon is the same as that seen with polymyxin B, which acts on the envelope of Gram-negative bacteria by disrupting and disorganizing the outer membrane [48]. Other studies have shown that some AMPs are able to promote membrane perturbations that ultimately lead to the lysis of the bacteria [53]. Finally, TEM images showed that the envelope of *E. coli* was affected by the presence of MPPN in a way similar to that observed in other cases [54]. MPPN caused the surface of *E. coli* to wrinkle, resulting in increased surface roughness.

In summary, MPPN is active against *E. coli* and *S. enterica*, but not *S. aureus*, and the main mechanisms of action involve membrane alterations (including disruption, disorganization and formation of pores) and inhibition of efflux pumps. The use of AMPs capable of inhibiting these efflux systems can be considered as a good tool to combat antimicrobial resistance, as they could be used alone or in combination with other conventional antibiotics to which bacteria have become resistant.

## Figures and Tables

**Table 1:** Characteristics of MPPN peptides.

Name ID	Peptide Name	Peptide Sequence	Type of Structure	Size, Amino Acids
PF-1	Prophenin-1	AFPPPNVPGPRFPPPNFPGPRFPPPNFPGPRFPPPNFPGPRFPPPNFPGPPFPPPIFPGPWFPPPPPFRPPPFGPPRFP	Type II poly-_L_-proline helix conformation	79
PF-2	Prophenin-2	AFPPPNVPGPRFPPPNVPGPRFPPPNFPGPRFPPPNFPGPRFPPPNFPGPPFPPPIFPGPWFPPPPPFRPPPFGPPRFP		79
PR-39	PR-39	RRRPRPPYLPRPRPPPFFPPRLPPRIPPGFPPRFPPRFP-NH2		39
PG-1	Protegrin-1	RGGRLCYCRRRFCVCVGR-NH2	Disulfide-bond-stabilized ϒ-core conformation	18
PG-2	Protegrin-2	RGGRLCYCRRRFCICV-NH2		16
PG-3	Protegrin-3	RGGGLCYCRRRFCVCVGR-NH2		18

NH2 denotes C-terminal amidation.

**Table 2:** Antimicrobial activity of MPPN (MIC and MBC).

	Gram-Negative					Gram-Positive	
	*E. coli* ATCC 25922	*A. baumannii* ATCC 17978	*P. aeruginosa* ATCC 27583	*K. pneumoniae* ATCC 28331	*S. enterica* ATCC 13076	*E. faecalis* ATCC 29212	*S. aureus* ATCC 29213
MIC (μg/mL)	68.7	>275	>275	>275	68.7	>275	>275
MBC (μg/mL)	137.5	>275	>275	>275	>275	>275	>275

MIC: minimal inhibitory concentration, MBC: minimal bactericidal concentration.

## Data Availability

The data used to support the findings of this study are available from the corresponding authors upon request.

## Supplementary Materials

The following supporting information can be downloaded at: https://www.mdpi.com/article/10.3390/microorganisms11082119/s1, Table S1: AO accumulation results.
